# The Editor’s Choice for Issue 2, Volume 8

**DOI:** 10.3390/ijns9020027

**Published:** 2023-05-05

**Authors:** Peter C. J. I. Schielen

**Affiliations:** Office-International Society for Neonatal Screening, Reigerskamp 273, NL-3607 HP Maarssen, The Netherlands; peter.schielen@isns-neoscreening.org

Volume 8, issue 2, consists of 15 papers, viewed by around 1500–2000 readers. The most read paper was ‘Towards Achieving Equity and Innovation in Newborn Screening across Europe’ [1], with over 3700 reads.

Remarkably, Natasha Heather and Dianne Webster of the New Zealand Neonatal Screening Program co-authored two contributions in this issue: one on SCID screening (with over 2500 views), reporting on almost 200,000 tested New Zealand newborns, and another on a protocol that can be used to improve the receipt of repeat samples after inadequate analysis or borderline test results [2,3].

In the Special Issue titled ‘Ethical and Psychosocial Aspects of Genomics in the Neonatal Period’, guest-edited by colleagues Dr. Lynn Bush and Dr Olaf Bodamer, twelve papers were published, three of which are published in this issue.

Here, I would like to highlight the contributions of Dr. Bush, Dr. Koehly, and co-authors, who described the experiences of families in caring for children with newborn screening (NBS)-related conditions, with special reference to the implications of genomic testing in population-based neonatal public health programs [4].

Neonatal screening is a system, not a test; within that system, confirmatory testing and (genetic) diagnoses answer some questions, but not all, and new questions are posed for caregivers and professionals, regarding prognostic uncertainty due to phenotypic variation. Thus, genetic diagnosis in newborn screening is often not the end of the so-called diagnostic odyssey, but is rather the beginning of what should be re-defined as a “diagnostic odyssey *continuum*”. The interpretation of a biochemical test in NBS is mostly straightforward, a positive result being defined by laboratory results below or above a cut-off value. Even in these simple cases, screening results at times may lead to such a diagnostic odyssey continuum. To add to these problematic outcomes, Dr. Bush, Dr. Koehly, and co-authors considered the increasing integration of genomic testing in neonatal screening. This would lead to new and more positive screening results; however, along with the benefits of early identification, there is added complexity, as the genetic results are harder to relate to neonatal pathogenicity. With growing uncertainty concerning the implications of the screening results, the timespan of the diagnostic odyssey continuum will not always be shortened and may be prolonged.

In shared decision making during this continuum, it is important to seek the voices of caregivers and to consider the lived experiences of families who are already caring for children with NBS-related conditions. Such perspectives are important to implement ethically nuanced screening policies. Hence, the authors set out to characterize the experiences of caregivers whose children’s illnesses are NBS-related conditions and to examine their experiences and perspectives in the context of this diagnostic odyssey continuum. Qualitative interviews were conducted using a mixed-methods approach for 169 participants from 77 families (which is a relatively large group for this type of study). 

The testimony delivered in the many citations of caregivers on how they experienced their “diagnostic odyssey *continuum*” should be read by all professionals in neonatal screening. They are an account of all the consequences, both positive, but certainly also detrimental, of a positive screening result. The authors presented their results in two domains, each illustrated with a telling quote from a caregiver, the many other quotes equally telling:

- Domain 1—medical management implications of a child diagnosed with NBS-related condition: “there’s so much, still, that’s unknown”;
- Domain 2—psychosocial implications of a child diagnosed with NBS-related condition: “We just don’t even have any clue what the outcome’s going to be like in this”.

The authors argue that even with current day neonatal screening, many of the needs of caregivers are not met, with questions remaining unanswered by health professionals. As more conditions are identified with population-based genome approaches, the gap between the needs of caregivers and what professionals can deliver will only become wider. Thus, significant improvements in the support for caregivers of a child with a positive neonatal test result are the prerequisite for the introduction of comprehensive genomic testing in neonatal screening. 

Such warnings have been issued in various forms across many contributions in this Special Issue ‘Ethical and Psychosocial Aspects of Genomics in the Neonatal Period’. Robert Currier delivered a comprehensive four-point summary of the ethical issues of genomic testing in neonatal screening [5], which are as follows:

1. DNA sequence results identify variants in the gene, but the inference of the possible disease state—whether early-onset, late-onset, or not penetrant—is difficult to predict for some conditions.
2. The interpretation of the clinical significance of the variants detected by sequencing relies on genomic databases that overrepresent the variants found in individuals of European ancestry and underrepresent the variants in individuals of other ancestries, hindering equitable explanations.
3. Genomic sequencing is currently more than two orders of magnitude more expensive than any current newborn screening test. Genomic sequencing in neonatal screening could divert resources from other responsibilities, including follow-up, diagnostic testing, and treatment.
4. Sequence data are intensely personal data for the newborn, which also has implications for the parents. This challenge to trust requires the newborn screening program to practice extreme transparency in how the DNA is used, whether residual DNA is stored, how the sequence data are generated, and how the results will be safeguarded for the future.

Additionally, a recent paper in *IJNS*, titled ‘Current State and Innovations in Newborn Screening: continuing to do good and avoid harm’ [6], stated that innovations in neonatal screening, especially genomic testing, should not undermine public confidence in acceptable and effective neonatal screening programs that have been developed over the past sixty years. 

Dr. Bush, Dr. Koehly, and co-authors identified eight recommendations to optimize ethically nuanced NBS expansion augmented with sequencing, one of which is:

“*The affordable and accessible provision of continuous, long-term counseling resources (genetic counseling, psychology, social work) for families to support, better understand, and cope with the diagnostic odyssey continuum and the evolution of the child’s condition […].*”

It may take many years to solidly establish even this one justified demand, let alone all eight. Until then, ethical considerations, as delivered by Bush et al., Currier, and many others, will hopefully prevent irresponsibly introducing screening that does not meet established ethical criteria.
